# Editorial: Comorbidities of adrenal-related endocrine disorders

**DOI:** 10.3389/fendo.2026.1801040

**Published:** 2026-02-26

**Authors:** Ivana Kraljevic, Emir Muzurovic

**Affiliations:** 1 School of Medicine, University of Zagreb, Zagreb, Croatia; 2 Department of Endocrinology, University Hospital Centre Zagreb, Zagreb, Croatia; 3 Faculty of Medicine, University of Montenegro, Podgorica, Montenegro; 4 Department of Internal Medicine, Endocrinology Section, Clinical Centre of Montenegro, Podgorica, Montenegro

**Keywords:** adrenal disorders, cardiometabolic risk, comorbidities, mild autonomous cortisol secretion (MACS), primary aldosteronism

## Introduction

Adrenal-related endocrine disorders represent a broad and heterogeneous group of conditions, ranging from mild hormonal abnormalities to life-threatening crises and malignancy. Traditionally, the primary focus in everyday clinical practice for patients with adrenal incidentaloma has been establishing the biochemical diagnosis and excluding malignancy, as these factors determine the need for intervention and the risk of serious clinical consequences (1). Multidisciplinary evaluation and clinical assessment are also recommended to ensure the patient’s overall health and comorbidities, rather than focusing solely on the adrenal lesion (2), because growing evidence suggests that an exclusively lesion-centered approach misses a significant part of the clinical burden (3–6).

Over the last few years, it has become clear that adrenal incidentalomas are frequently associated with vertebral fractures, hypertension, type 2 diabetes mellitus, dyslipidemia, obesity, and increased cardiovascular risk (3–8). These comorbidities are particularly pronounced in patients with mild autonomous cortisol secretion (MACS), which is present in up to 30–50% of adrenal incidentaloma cases and is the most frequent hormonal abnormality in this population (3, 4). The studies included in this Research Topic illustrate how the consequences of adrenal disease unfold over time, involve multiple organ systems, and are not resolved by normalizing hormone levels alone.

## Adrenal incidentalomas: recognition and referral gaps

The widespread use of cross-sectional imaging has led to adrenal incidentalomas being a common finding on radiology reports (7). However, this frequent imaging diagnosis does not always result in a structured endocrine work-up. In a large tertiary cohort from Lebanon, Jambart et al. reported a prevalence of adrenal incidentalomas of around 7%, yet only 5.4% of affected patients were referred for endocrinological evaluation. This discrepancy illustrates how health systems respond—or fail to respond—to a “routine” radiological finding. For many patients, the adrenal lesion was documented once and then effectively ignored. Without standardized hormonal screening, risk assessment, and follow-up, a potential opportunity to identify early clinically relevant adrenal hormone excess and related comorbidities is lost.

## Primary aldosteronism: beyond blood pressure

Primary aldosteronism (PA) is the most frequent cause of secondary hypertension, yet it remains under-recognized and often suboptimally treated (9). The persistent perception of PA as a rare, highly specialized condition further limits the implementation of systematic screening and timely management. Several articles in this Research Topic address different aspects of this problem, including the prediction of outcomes, the development of novel diagnostic tools, and the characterization of associated endocrine comorbidities. Wang et al. developed a predictive nomogram to estimate the likelihood of incomplete clinical success after unilateral adrenalectomy. This mirrors what clinicians commonly observe in practice: normalization of aldosterone does not necessarily result in immediate or complete improvement in blood pressure or metabolic control. In another study, Wang et al. evaluated sublingual microcirculatory dysfunction as a non−invasive screening tool for PA. They found a strong discriminatory capacity, suggesting that vascular functional assessment may provide complementary information to standard biochemical testing. At the molecular level, Makhnov et al. demonstrated that specific circulating microRNA profiles can distinguish PA from essential hypertension, illustrating the potential of molecular signatures and machine learning to refine endocrine diagnostics.

Beyond blood pressure and potassium levels, PA is increasingly viewed as a multisystem endocrine disorder (10). A systematic review and meta-analysis by Zhang et al. reported a higher prevalence of thyroid disease among patients with PA, particularly thyroid nodules, whereas associations with other thyroid conditions, such as hypothyroidism, hyperthyroidism, thyroiditis, or thyroid cancer, were not observed. Taken together, these studies support a more integrated endocrine assessment in PA, shifting the focus from isolated blood pressure management toward a multi-organ view that combines clinical, biochemical, imaging, vascular, and molecular data.

## Clinical impact of cortisol excess and insufficiency

The spectrum of cortisol dysregulation ranges from MACS to overt adrenal insufficiency. Mansour et al. investigated patients with PA and concomitant MACS and showed that even modest cortisol excess is associated with reduced volumetric bone mineral density at the lumbar spine, reinforcing previous evidence that “mild” cortisol excess affects skeletal health even in the absence of overt Cushingoid features (11). At the other end of the spectrum, Qiu et al. analyzed adrenal crisis in adults with glucocorticoid-induced adrenal insufficiency, identified clinical and laboratory predictors, and confirmed an association with increased mortality. The study illustrates how the risk profile shifts from complications of hormone excess to complications of deficiency, while the overall impact on morbidity and survival remains considerable.

These data highlight the dual burden of cortisol excess and deficiency. Practically, they support the need for careful long-term follow-up, timely adjustment of glucocorticoid therapy, and heightened awareness of “borderline” cortisol states that might otherwise be underestimated. For clinicians, the key message is that subtle cortisol imbalance can carry clinically relevant risk and should not be regarded as a purely biochemical finding.

## Lifelong burden of congenital adrenal hyperplasia

Congenital adrenal hyperplasia (CAH) due to 21−hydroxylase deficiency is a lifelong condition with a complex clinical course. Two contributions in this Research Topic address complementary aspects of its long−term burden. Aldalaan et al. reported outcomes from a tertiary cohort of adults with CAH and found a high prevalence of metabolic, reproductive, and growth-related complications that persist into later life, reflecting the cumulative impact of chronic hormonal imbalance, prolonged glucocorticoid exposure, and the difficulty of maintaining adequate control over decades. In parallel, Hubska et al. reviewed endothelial dysfunction and emerging vascular biomarkers in CAH, linking vascular changes and increased cardiovascular risk to both the underlying disease and its treatment.

Overall, these studies underscore that CAH is not only a disorder of cortisol and androgen synthesis in early life but also a condition with sustained, multi-organ consequences (12). Cardiometabolic risk, reproductive health, growth, and bone status may all be affected, and the vascular system appears particularly vulnerable (12). For clinicians, this implies that management should be planned with a lifelong perspective, including regular re−evaluation of therapy, proactive screening for comorbidities, and structured transition from pediatric to adult endocrine care.

## Steroidogenesis and developmental metabolic profiles

Lorek et al. present an alternative perspective by examining steroidogenesis during the neonatal period. Using comprehensive steroid profiling, they identified distinct metabolomic fingerprints in preterm and term neonates. Although this study does not address a classical adrenal disorder, it illustrates how adrenal steroid production is tightly linked to early postnatal adaptation and short−term outcomes. Differences in metabolomic profiles may help explain why some neonates are particularly vulnerable to complications and could, in the future, guide earlier and more targeted neonatal interventions.

This work suggests that adrenal biology and its interaction with developmental metabolic pathways may influence long-term risk, even if clinical consequences are not immediately apparent. In this sense, the study serves as a reminder that adrenal-related comorbidities are not limited to adulthood.

## Clinical case insights from a real-world

Clinical presentations often diverge from classic textbook patterns, as illustrated by the case report by Zhang et al. The authors describe a patient with hypertension, hypokalemia, and bilateral adrenal adenomas, highlighting the diagnostic and therapeutic challenges that arose. The case underscores the difficulty of reconciling overlapping imaging findings, biochemical data, and clinical priorities, particularly when multiple adrenal lesions and potential pathogenic mechanisms coexist.

## Conclusion and future directions

Although the adrenal-related conditions covered in this Research Topic differ in presentation, underlying mechanisms, and treatment, they share a common theme: adrenal disorders have consequences that extend well beyond isolated hormonal abnormalities. Diagnostic delays, multisystem comorbidities, and the demands of long-term management recur across the included studies. Contributions on adrenal incidentalomas, PA, cortisol excess and insufficiency, CAH, neonatal steroidogenesis, and complex case reports provide a broad, clinically grounded view of how adrenal disease affects multiple organ systems and interacts with healthcare delivery.

Looking forward, future research should link endocrine dysregulation more directly to outcomes that matter to patients—survival, functional status, and quality of life—rather than focusing solely on hormone levels (13). Integrating molecular tests, functional assessments, imaging, and predictive tools into routine clinical practice can improve risk stratification and enable earlier, more individualized interventions. For clinicians, the key message is that recognizing and managing comorbidities should be considered an integral part of adrenal endocrinology practice.
